# Resistance in Soybean Against Infection by *Phakopsora pachyrhizi* Is Induced by a Phosphite of Nickel and Potassium

**DOI:** 10.3390/plants13223161

**Published:** 2024-11-11

**Authors:** Bianca Apolônio Fontes, Leandro Castro Silva, Bárbara Bezerra Menezes Picanço, Aline Vieira Barros, Isabela Maria Grossi Leal, Leonardo Packer Quadros, Fabrício Ávila Rodrigues

**Affiliations:** Laboratório da Interação Planta-Patógeno, Departamento de Fitopatologia, Universidade Federal de Viçosa, Viçosa 36570-900, MG, Brazil; biancafontes@gmail.com (B.A.F.); leandrocsilva1989@gmail.com (L.C.S.); barbara.picanco@gmail.com (B.B.M.P.); alinevieiradebarros@gmail.com (A.V.B.); isabela.leal@ufv.br (I.M.G.L.); leonardo.quadros@ufv.br (L.P.Q.)

**Keywords:** *Glycine max*, antioxidative metabolism, biotrophic pathogen, plant defense reactions, photosynthesis, rust

## Abstract

Soybean (*Glycine max* (L.) Merr.) is one of the most profitable crops among the legumes grown worldwide. The occurrence of rust epidemics, caused by *Phakopsora pachyrhizi*, has greatly contributed to yield losses and an abusive use of fungicides. Within this context, this study investigated the potential of using a phosphite of nickel (Ni) and potassium (K) [referred to as induced resistance (IR) stimulus] to induce soybean resistance against infection by *P. pachyrhizi*. Plants were sprayed with water (control) or with IR stimulus and non-inoculated or inoculated with *P. pachyrhizi*. The germination of urediniospores was greatly reduced in vitro by 99% using IR stimulus rates ranging from 2 to 15 mL/L. Rust severity was significantly reduced from 68 to 78% from 7 to 15 days after inoculation (dai). The area under the disease progress curve significantly decreased by 74% for IR stimulus-sprayed plants compared to water-sprayed plants. For inoculated plants, foliar concentrations of K and Ni were significantly higher for IR stimulus treatment than for the control treatment. Infected and IR stimulus-sprayed plants had their photosynthetic apparatus (a great pool of photosynthetic pigments, and lower values for some chlorophyll *a* fluorescence parameters) preserved, associated with less cellular damage (lower concentrations of malondialdehyde, hydrogen peroxide, and anion superoxide) and a greater production of phenolics and lignin than plants from the control treatment. In response to infection by *P. pachyrhizi*, defense-related genes (*PAL2.1*, *PAL3.1*, *CHIB1*, *LOX7*, *PR-1A*, *PR10*, *ICS1*, *ICS2*, *JAR*, *ETR1*, *ACS*, *ACO*, and *OPR3*) were up-regulated from 7 to 15 dai for IR stimulus-sprayed plants in contrast to plants from the control treatment. Collectively, these findings provide a global picture of the enhanced capacity of IR stimulus-sprayed plants to efficiently cope with fungal infection at both biochemical and physiological levels. The direct effect of this IR stimulus against urediniospores’ germination over the leaf surface needs to be considered with the aim of reducing rust severity.

## 1. Introduction

Due to it possessing the highest content of protein and oil, soybean (*Glycine max* (L.) Merr.) became the most profitable crop grown worldwide, and plays a pivotal role in livestock, human food, and biodiesel production [1]. The biotrophic fungus *Phakopsora pachyrhizi* H. Sydow and P. Sydow, the causal agent of soybean rust (SR), is among the most destructive pathogens affecting soybean production worldwide [2,3]. The infection process of this fungus starts with the deposition of urediniospores on the abaxial leaf surface and, under favorable environmental conditions, their germination takes place to give rise to a germ tube, from which a melanized appressorium is formed to allow for penetration into the epidermal cell [4]. Small yellowish-brown lesions, restricted by the main veins in the leaves, are formed as a result of a successful infection process [4]. On infected leaves, many necrotic lesions formed on the leaflets, with discreet chlorosis around them, cause the premature defoliation and earlier maturation of organs from the plants, resulting, therefore, in significant yield losses [4,5,6].

In Brazil, the management of SR is mainly achieved by spraying fungicides composed of molecules with different modes of action (e.g., triazole, strobilurin, and carboxamide groups, or a mixture of triazoles with strobilurins) [5]. A total of 215 products (e.g., contact and systemic fungicides formulated using different active ingredients or their mixtures) have been registered in the Ministry of Agriculture, Livestock, and Food Supply (MALF) for the control of SR [7]. There is no federal legislation or official recommendation by the MALF limiting the amount of fungicide spray as well as the use of active ingredients during the soybean growing season. Some cultural practices (e.g., preference for early-maturing cultivars, paying attention to the appearance of earlier rust lesions, adopting a period of not growing soybean in the off-season, and following a specific sowing date) are other strategies for SR management [5,8]. Unfortunately, soybean cultivars exhibiting race-specific resistance or with higher levels of non-race resistance against SR have not yet been made available to farmers [9,10,11]. It is important to point out that the continuous use of systemic fungicides from the same chemical group can lead to the selection of resistant or less-sensitive individuals in the population of *P. pachyrhizi* resulting, therefore, in low efficacy in disease control [8]. Nowadays, populations of *P. pachyrhizi* with less sensitivity to the current molecules in the fungicides have become an actual concern of many agricultural chemical companies and growers that own technologically modified farms that use at least four sprays of fungicide during the soybean growing season [5,8]. Considering the occurrence of severe SR epidemics in each soybean growing season and the increased concern regarding the loss of efficiency of the fungicides used to hamper the infection process of *P. pachyrhizi* that will slow the disease progress rate, the use of resistance inducers may become a plausible alternative towards more sustainable agriculture. In this regard, reduction in the production costs and less impact to the environment and human due to a reduction in the number of fungicide sprays, or intercalating then with resistance inducers during the crop season, highlight the adoption of using the resistance inducers in soybean production.

Induced resistance (IR) is a well-known phenomenon in plants after being stimulated by abiotic or biotic IR stimuli, resulting in a physiological state of greater defensive capacity against several pathogens [12,13]. Systemic acquired resistance (SAR) and induced systemic resistance (ISR) are the two types of IR taking place in plants, countering infection by pathogens of different lifestyles [14,15,16]. The SAR involves a rapid, systemic, and long-lasting defense response to impair the colonization of plant tissues by pathogens [16]. The SAR is activated after plants are exposed to different IR stimuli and is closely linked to salicylic acid (SA) production and the expression of pathogenesis-related (PR) proteins [15,17]. Several metabolites (e.g., azelaic acid, pipecolic acid, glycerol-3-phosphate, SA methyl ester, and dehydroabietinal) are involved in the SAR signaling pathway in plants [18,19], while ISR is activated by plant growth-promoting rhizobacteria or by some IR stimuli, with the signaling pathway being mediated by jasmonic acid in collaboration with the ethylene [14,20]. The literature emphasizes the importance of using different IR stimuli (acibenzolar-S-methyl, harpin protein-derived peptides, nickel, silicon, *Bacillus subtilis*, saccharin, azelaic acid, hexanoic acid, salicylic acid, inorganic salts, phosphites) to induce the defense responses of plants, aiming to obtain a satisfactory level of disease control in very profitable crops [3,21,22,23,24,25,26,27]. The use of phosphites to control various diseases in soybean, including SR, is well documented in the literature [3,28,29,30]. Under field conditions, soybean plants sprayed with potassium phosphite showed reduced SR severity, increased polyphenoloxidase activity, and a higher expression of genes such as *GmAOX2a* (encoding an alternative oxidase) and those encoding for PR proteins (*PR1*, *PR2*, *PR3*, *PR4*, *PR5*, and *PR8*) [31]. Moreover, experiments carried out under greenhouse conditions showed the potential of formulations of phosphites containing copper, manganese or potassium to potentiate soybean resistance against infection by *P. pachyrhizi* as a result of increased activities of chitinase, phenylalanine ammonia-lyase, and *β*-1,3-glucanase [3,29,30,32]. However, new formulations of phosphite combined with nutrients other than copper, manganese or potassium need to be tested to determine their efficiency in controlling the foliar disease affecting profitable crops.

Considering the economic importance of soybean for the global economy and the major threat that SR imposes on food security, the present study hypothesized that the spraying of soybean plants with a different formulation of phosphite, containing the micronutrient nickel combined with potassium, could potentiate their resistance against infection by *P. pachyrhizi*. This hypothesis was tested by performing several analyses at the biochemical, physiological, and molecular levels that included the examination of the photosynthetic apparatus, the antioxidative metabolism, and the host defense responses in plants that were non-sprayed or sprayed with the phosphite and non-inoculated or inoculated with *P. pachyrhizi*.

## 2. Results

### 2.1. Analysis of Variance

The effect of control (water) and IR stimulus treatments [named as products (P)] on urediniospores’ germination was analyzed by one-way ANOVA. The factor P was significant (*p* < 0.001) for urediniospores’ germination. The response of all variables and parameters to P, plant inoculation (PI), and the P × PI interaction was analyzed by a two-way ANOVA. The factors P and PI, as well as the P × PI interaction, were significant (*p* ≤ 0.05) for most of the variables and parameters studied (Table 1).

### 2.2. Germination of Urediniospores In Vitro

**Figure 1:**
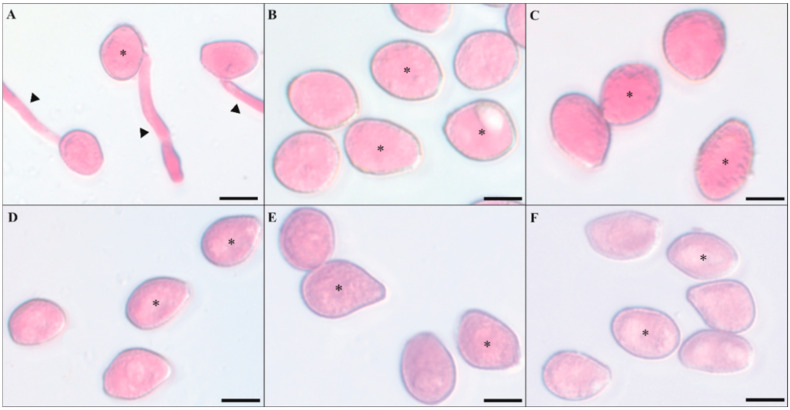
Aspects of the germination of urediniospores from *Phakopsora pachyrhizi* in glass slides containing different rates of the induced resistance (IR) stimulus (2, 5, 7, 10, and 15 mL L^−1^, respectively) to (**B**–**F**). The control treatment corresponded to urediniospores’ suspension without IR stimulus (**A**). Germ tube (arrowheads) and urediniospores (*). Scale bars = 5 μm.

**Figure 2:**
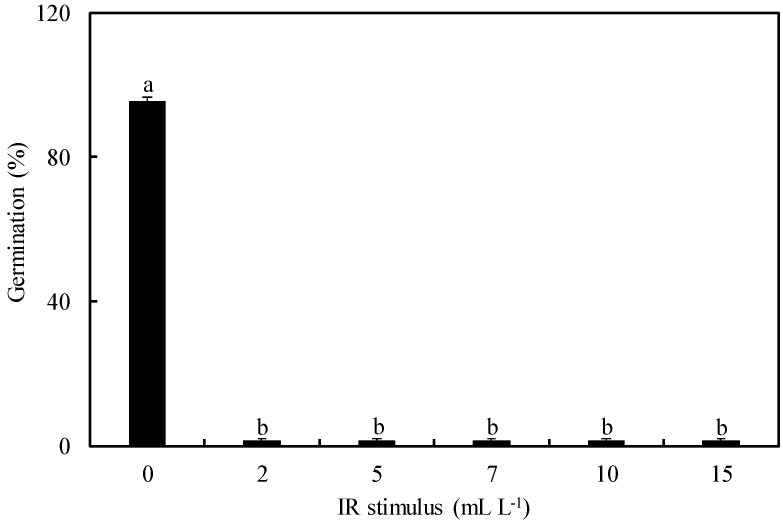
The germination of urediniospores from *Phakopsora pachyrhizi* in Petri dishes containing agar-agar medium non-amended (control) or amended with different rates of induced resistance (IR) stimulus. Means from each treatment followed by different letters are significantly different (*p* ≤ 0.05) according to Tukey’s test. Bars represent the standard error of the means.

The urediniospores from *P. pachyrhizi* did not germinate when exposed to the IR stimulus rates ranging from 2 to 15 mL/L compared to the control treatment (Figure 1A–F). Urediniospores’ germination was significantly reduced by 99% for IR stimulus with rates ranging from 2 to 15 mL/L compared to the control treatment (Figure 2).

### 2.3. Rust Symptoms and Severity, AUDPC, and Observations at the SEM

**Figure 3:**
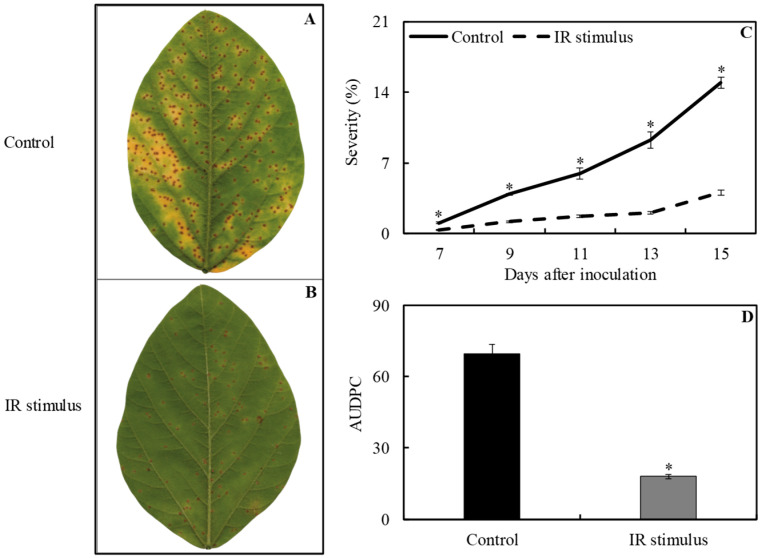
The symptoms (chlorosis and necrosis) (**A**,**B**) and severity (**C**) of soybean rust, as well as the area under disease progress curve (AUDPC) (**D**) for soybean plants sprayed with water (control) or with induced resistance (IR) stimulus. Means for control and IR stimulus followed by an asterisk (*), at each evaluation time, (**C**) or between these treatments for AUDPC followed by * (**D**), are significantly different (*p* ≤ 0.05) according to an *F* test. Bars represent the standard error of the means.

**Figure 4:**
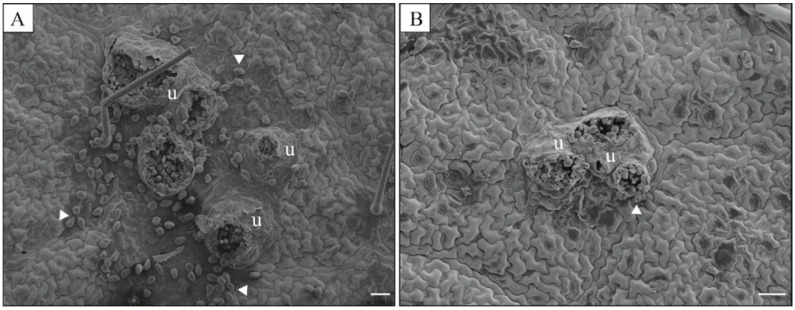
Scanning electron micrographs of the abaxial leaf surface of soybean plants at 15 days after inoculation with *Phakopsora pachyrhizi* and sprayed with water (control) (**A**) or with induced resistance stimulus (**B**). Uredia (u) and urediniospores (arrowheads). Scale bars = 50 μm.

Necrotic lesions containing many uredinia were abundant on the leaflets of plants from the control treatment in contrast to the leaflets of IR-stimulus-sprayed plants (Figure 3A,B). Rust severity was significantly reduced by 68, 70, 72, 78, and 73%, respectively, at 7, 9, 11, 13, and 15 dai for IR-stimulus-sprayed plants compared to plants from the control treatment (Figure 3C). The AUDPC significantly decreased by 74% for IR-stimulus-sprayed plants compared to plants from the control treatment (Figure 3D). The uredinia formed in the leaflets of IR-stimulus-sprayed plants were smaller and more compact than those observed on the leaflets of plants from the control treatment (Figure 4A,B). Many urediniospores were found inside the uredinia formed on necrotic lesions in the leaflets of plants from the control treatment (Figure 4A).

### 2.4. Foliar Concentrations of Ni and K

**Figure 5:**
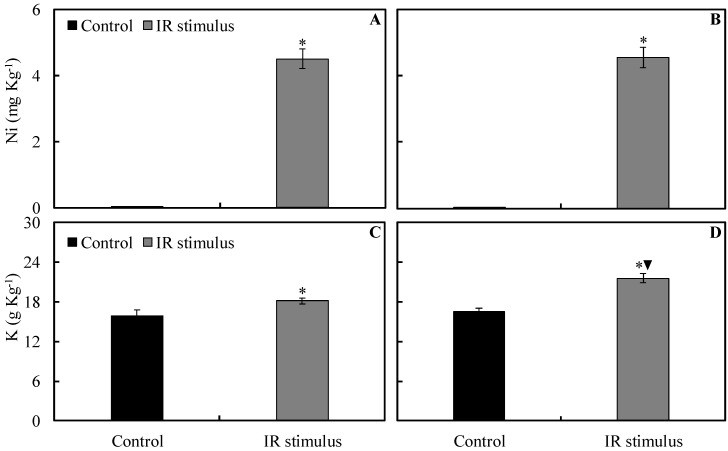
The foliar concentration of nickel (Ni) (**A**,**B**) and potassium (K) (**C**,**D**) for soybean plants non-inoculated (NI) (**A**,**C**) or inoculated (I) (**B**,**D**) with *Phakopsora pachyrhizi* and sprayed with water (control) or with induced resistance (IR) stimulus. Means for control and IR stimulus treatments followed by an asterisk (*) and means for NI and I plants followed by an inverted triangle (▼) are significantly different (*p* ≤ 0.05) according to an *F* test. Bars represent the standard error of the means.

The foliar Ni concentration significantly increased for non-inoculated and inoculated plants sprayed with the IR stimulus compared to non-inoculated and inoculated plants from the control treatment (Figure 5A,B). The foliar K concentration was significantly higher, by 14 and 31%, respectively, for non-inoculated and inoculated plants sprayed with IR stimulus compared to non-inoculated and inoculated plants from the control treatment. For IR stimulus treatment, the foliar concentration of K significantly increased by 19% for inoculated plants compared to non-inoculated plants (Figure 5C,D).

### 2.5. Imaging and Quantification of Chl a Fluorescence Parameters

**Figure 6:**
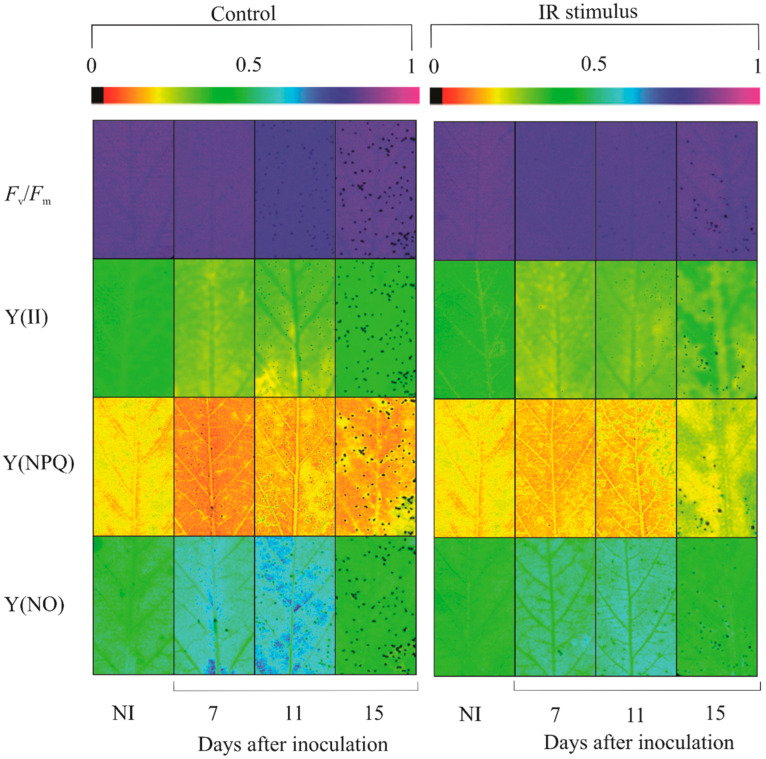
Images of chlorophyll *a* fluorescence parameters: maximum photosystem II quantum yield (*F*_v_/*F*_m_), effective photosystem II quantum yield (Y(II)), quantum yield of regulated energy dissipation [Y(NPQ)], and quantum yield of non-regulated energy dissipation [Y(NO)], determined in the leaflets of soybean plants sprayed with water (control) or with induced resistance (IR) stimulus and non-inoculated (NI) or at 7, 11, and 15 days after inoculation with *Phakopsora pachyrhizi*.

There was no alteration in the images of Chl *a* fluorescence parameters for non-inoculated plants regardless of treatments and sampling times (Figure 6). Remarkable damage to photosynthetic apparatus occurred for the inoculated plants from the control treatment compared to inoculated plants sprayed with IR stimulus at 15 dai based on the darker areas in the images corresponding to *F*_v_/*F*_m_, Y(II), Y(NPQ), and Y(NO) parameters (Figure 6).

**Figure 7:**
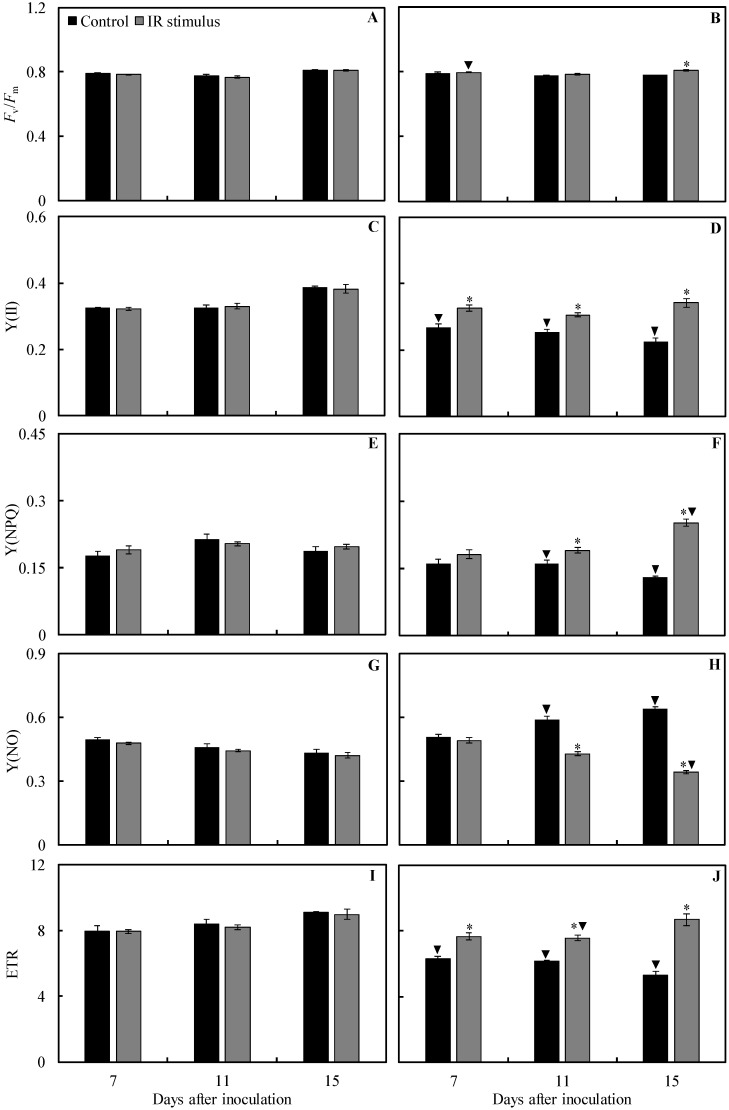
The quantification of chlorophyll *a* fluorescence parameters: maximum photosystem II quantum yield (*F*_v_/*F*_m_) (**A**,**B**), effective photosystem II quantum yield [Y(II)] (**C**,**D**), quantum yield of regulated energy dissipation [Y(NPQ)] (**E**,**F**), quantum yield of non-regulated energy dissipation [Y(NO)] (**G**,**H**), and electron transport rate (ETR) (**I**,**J**) in the leaflets of soybean plants sprayed with water (control) and with induced resistance (IR) stimulus and non-inoculated (NI) (**A**,**C**,**E**,**G**,**I**) or inoculated (I) (**B**,**D**,**F**,**H**,**J**) with *Phakopsora pachyrhizi*. Means for control and IR stimulus treatments followed by an asterisk (*) and means for NI and I plants followed by an inverted triangle (▼), at each evaluation time, are significantly different (*p* ≤ 0.05) according to an *F* test. Bars represent the standard error of the means.

There was no significant difference between non-inoculated and inoculated plants from the control treatment for *F*_v_/*F*_m_ regardless of the evaluation time. The values for Y(II) (17, 22, and 42% at 7, 11, and 15 dai, respectively), Y(NPQ) (26 and 30% at 11 and 15 dai, respectively), and ETR (21, 27, and 42% at 7, 11, and 15 dai, respectively) were significantly lower, while the values for Y(NO) (28 and 48% at 11 and 15 dai, respectively) were significantly higher for inoculated plants in comparison to non-inoculated plants (Figure 7A–J). There was no significant difference between non-inoculated and inoculated plants for IR stimulus treatment for Y(II) regardless of the evaluation time. The values for *F*_v_/*F*_m_ (1.4% at 7 dai) and Y(NPQ) (28% at 15 dai) were significantly higher, while the values for Y(NO) (18% at 15 dai) and ETR (8% at 11 dai) were significantly lower for inoculated plants compared to non-inoculated plants (Figure 7A–J).

For non-inoculated plants, there was no significant difference for any of the treatments regardless of the evaluation time (Figure 7A,C,E,G,I). For inoculated plants, the values for *F*_v_/*F*_m_ (1% at 11 dai), Y(II) (21, 20, and 51% at 7, 11, and 15 dai, respectively), Y(NPQ) (19 and 92% at 11 and 15 dai, respectively), and ETR (22, 23, and 63% at 7, 11, and 15 dai, respectively) were significantly higher, while the values for Y(NO) (27 and 46% at 11 and 15 dai, respectively) were significantly lower for IR stimulus treatment compared to the control treatment (Figure 7B,D,F,H,J).

### 2.6. Photosynthetic Pigment Concentration

**Figure 8:**
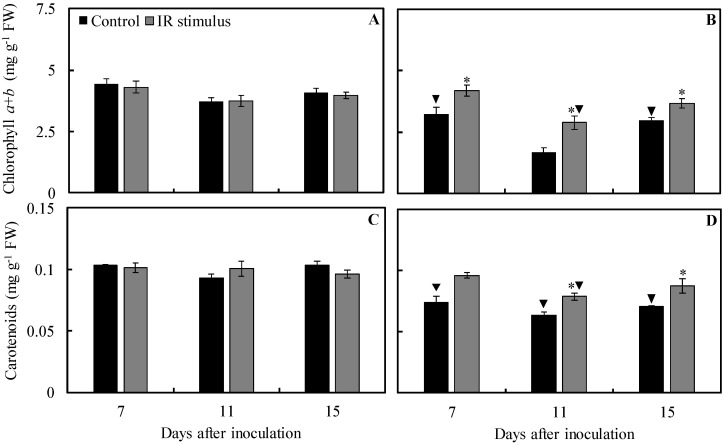
The concentrations of chlorophyll *a* + *b* (Chl *a* + *b*) (**A**,**B**) and carotenoids (**C**,**D**) determined in the leaflets of soybean plants sprayed with water (control) or with induced resistance (IR) stimulus and non-inoculated (NI) (**A**,**C**) or inoculated (I) (**B**,**D**) with *Phakopsora pachyrhizi*. Means for control and IR stimulus treatments followed by an asterisk (*) and means for NI and I plants followed by an inverted triangle (▼), at each evaluation time, are significantly different (*p* ≤ 0.05) according to an *F* test. Bars represent the standard error of the means. FW = fresh weight.

For the control treatment, concentrations of Chl *a* + *b* (30 and 27% at 7 and 15 dai, respectively) and carotenoids (29, 32, and 32% at 7, 11, and 15 dai, respectively) were significantly lower for inoculated plants compared to non-inoculated plants (Figure 8A–D). For IR stimulus treatment, concentrations of Chl *a* + *b* (23% at 11 dai) and carotenoids (22% at 11 dai) were significantly lower for inoculated plants in comparison to non-inoculated plants (Figure 8A–D).

The concentration of Chl *a* + *b* and carotenoids for non-inoculated plants was not significantly affected by any of the treatments, regardless of the evaluation time (Figure 8A,C). For inoculated plants, the concentrations of Chl *a* + *b* (29, 71, and 23% at 7, 11, and 15 dai, respectively) and carotenoids (25 and 24% at 11 and 15 dai, respectively) were significantly higher for IR stimulus treatment in comparison to the control treatment (Figure 8B,D).

### 2.7. Histochemical Analysis

**Figure 9:**
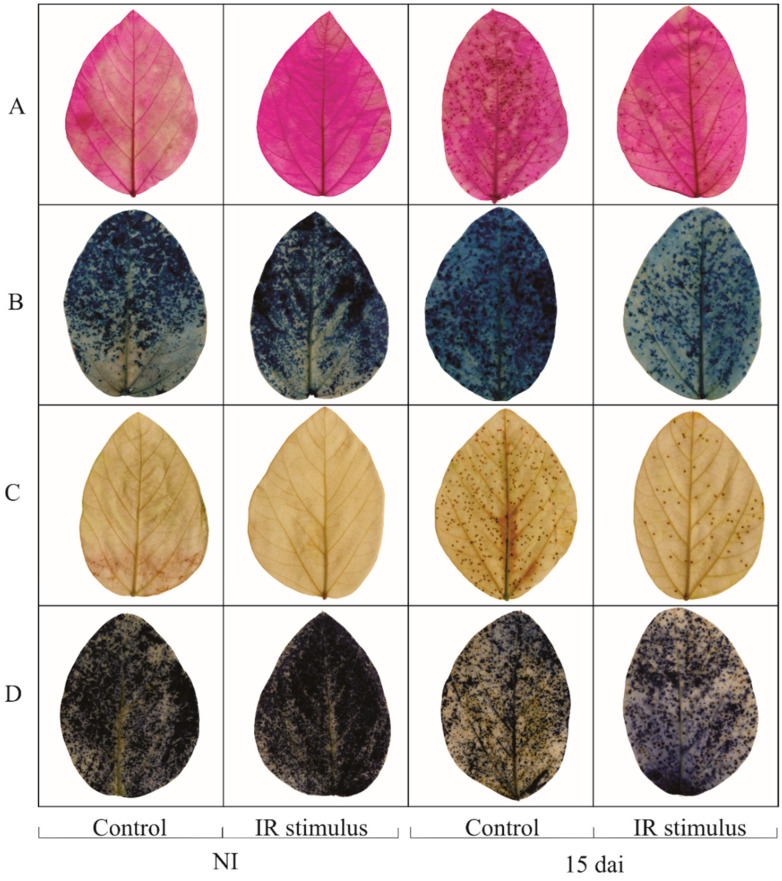
The histochemical detection of lipid peroxidation (**A**), membrane damage (**B**), hydrogen peroxide (**C**), and superoxide anion radical (**D**) on the leaves of soybean plants non-inoculated (NI) or at 15 days after inoculation (dai) with *Phakopsora pachyrhizi*, previously sprayed with water (control) or with induced resistance (IR) stimulus.

The spraying of IR stimulus did not cause any physiological perturbation to the leaflets of non-inoculated plants, as evidenced by the absence of staining for lipid peroxidation, membrane damage, H_2_O_2_, and O_2_^•−^ compared to leaflets of plants from the control treatment (Figure 9A–D). Lipid peroxidation, membrane damage, and depositions of H_2_O_2_ and O_2_^•−^ (brown and blue colors, respectively) were less intense in the leaflets of plants sprayed with the IR stimulus than on the leaflets of plants from the control treatment at 15 dai (Figure 9A–D).

### 2.8. Concentrations of MDA, H_2_O_2_, and O_2_^•−^

**Figure 10:**
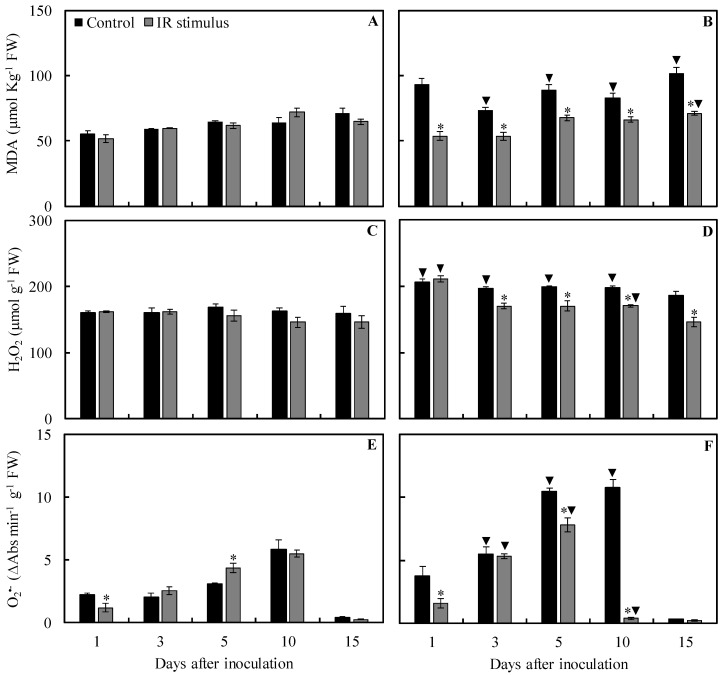
The concentration of malondialdehyde (MDA) (**A**,**B**), hydrogen peroxide (H_2_O_2_) (**C**,**D**), and superoxide anion radical (O_2_^•−^) (**E**,**F**) determined in the leaflets of soybean plants sprayed with water (control) or with induced resistance (IR) stimulus and non-inoculated (**A**,**C**,**E**) or inoculated (**B**,**D**,**F**) with *Phakopsora pachyrhizi*. Means for control and IR stimulus treatments followed by an asterisk (*) and means for NI and I plants followed by an inverted triangle (▼), at each evaluation time, are significantly different (*p* ≤ 0.05) according to an *F* test. Bars represent the standard error of the means. FW = fresh weight.

For the control treatment, concentrations of MDA (23, 38, 30, and 44% at 3, 5, 10, and 15 dai, respectively), H_2_O_2_ (29, 23, 18, and 21% at 1, 3, 5, and 10 dai, respectively), and O_2_^•−^ (175, 239, and 86% at 3, 5, and 10 dai, respectively) were significantly higher for inoculated plants in comparison to non-inoculated plants (Figure 10A–F). For IR stimulus treatment, concentrations of MDA (10% at 15 dai), H_2_O_2_ (31 and 17% at 1 and 10 dai, respectively), and O_2_^•−^ (112 and 81% at 3 and 5 dai, respectively) were significantly higher, while the concentration of O_2_^•−^ (93% at 10 dai) was significantly lower for inoculated plants compared to non-inoculated plants (Figure 10A–F).

The concentrations of MDA and H_2_O_2_ for non-inoculated plants were not affected by any of the treatments regardless of the evaluation time. The concentration of O_2_^•−^ was significantly lower by 47% at 1 dai and significantly higher by 40% at 5 dai for IR stimulus treatment compared to the control treatment (Figure 10A,C,E). For inoculated plants, the concentrations of MDA (42, 27, 24, 20, and 30% at 1, 3, 5, 10, and 15 dai, respectively), H_2_O_2_ (14, 14, 14, and 22% at 3, 5, 10, and 15 dai, respectively), and O_2_^•−^ (58, 26, and 97% at 1, 5, and 10 dai, respectively) were significantly lower for IR stimulus treatment in comparison to the control treatment (Figure 10B,D,F).

### 2.9. Concentrations of TSP and LTGA Derivatives

**Figure 11:**
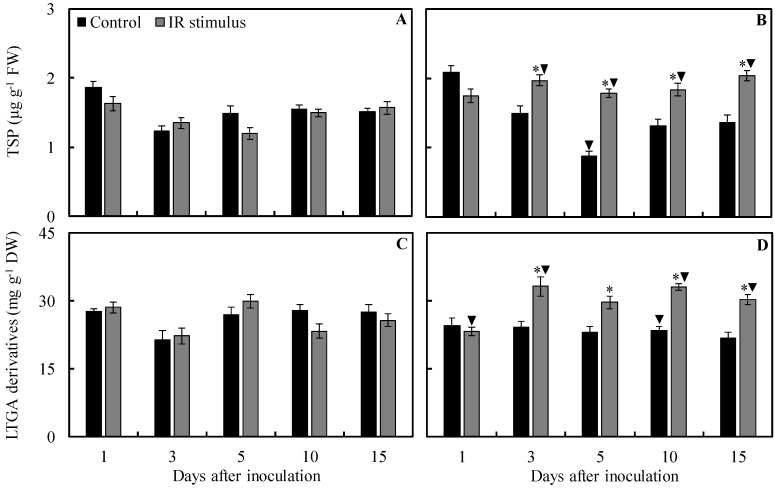
The concentration of total soluble phenolics (TSP) and lignin-thioglycolic acid (LTGA) derivatives determined in the leaflets of soybean plants sprayed with water (control) or with induced resistance (IR) stimulus and non-inoculated (**A**,**C**) or inoculated (**B**,**D**) with *Phakopsora pachyrhizi*. Means for control and IR stimulus treatments followed by an asterisk (*) and means for NI and I plants followed by an inverted triangle (▼), at each evaluation time, are significantly different (*p* ≤ 0.05) according to an *F* test. Bars represent the standard error of the means. FW and DW = fresh weight and dry weight, respectively.

For the control treatment, the concentrations of TSP (41% at 5 dai) and LTGA derivatives (16% at 10 dai) were significantly lower for inoculated plants in comparison to non-inoculated plants (Figure 11C,D). For IR stimulus treatment, the concentrations of TSP (46, 49, 23, and 30% at 3, 5, 10, and 15 dai, respectively) and LTGA derivatives (49, 42, and 18% at 3, 10, and 15 dai, respectively) were significantly higher, while the concentration of LTGA derivatives (19% at 1 dai) was significantly lower for inoculated plants in comparison to non-inoculated plants (Figure 11A–D).

The concentrations of TSP and LTGA derivatives were not affected by any of the treatments for non-inoculated plants regardless of evaluation time (Figure 11A,C). Concentrations of TSP (31, 103, 40, and 49% at 3, 5, 10, and 15 dai, respectively) and LTGA derivatives (37, 28, 41, and 40% at 3, 5, 10, and 15 dai, respectively) for inoculated plants were significantly higher for the IR stimulus treatment compared to the control treatment (Figure 11B,D).

### 2.10. Gene Expression

#### 2.10.1. Comparing Control and IR Stimulus Treatments for Non-Inoculated Plants

**Figure 12:**
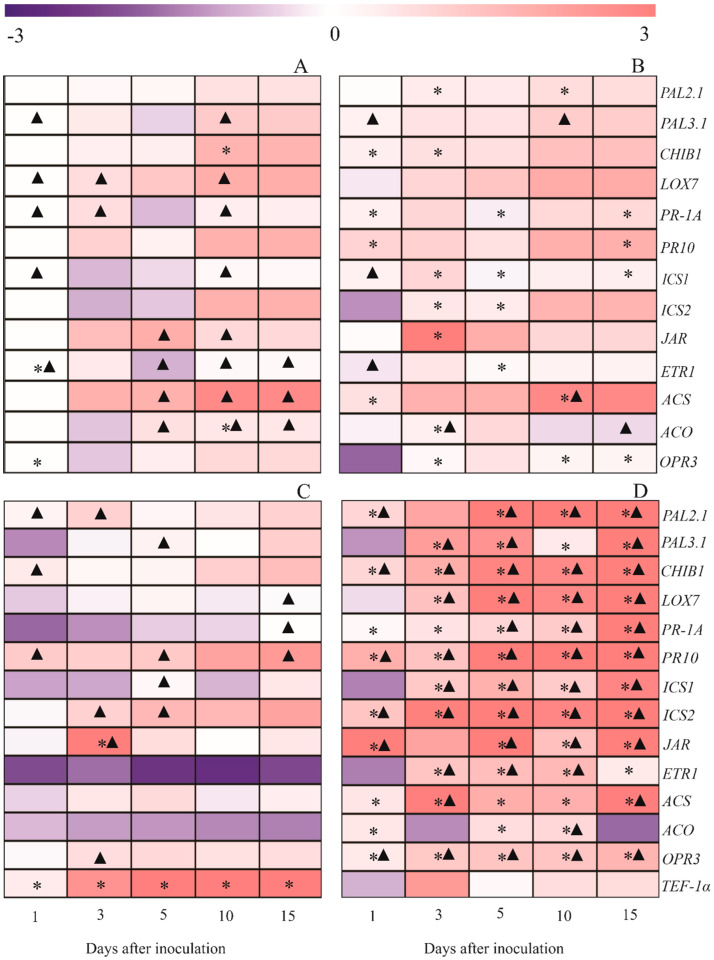
The expression profile of genes determined in the leaflets of soybean plants sprayed with water (control) (**A**,**C**) or with induced resistance (IR) stimulus (**B**,**D**) and non-inoculated (NI) (**A**,**B**) or inoculated (I) (**C**,**D**) with *Phakopsora pachyrhizi*. Color cells ranging from purple (−3.0) to red (+3.0) represent the relative transcript levels for the genes studied. The amplification of glyceraldehyde 3-phosphate dehydrogenase (*GAPDH*) and Ubiquitin-3 (*UBIQ*) genes from soybean was used as an internal control for data normalization. Fold changes were calculated based on transcript level for NI plants from the control treatment at 1 day after inoculation (dai), except for *TEF-1α*. In this case, transcript levels of *TEF-1α* for I plants from the control treatment at 1 dai were used in the calculation. Four biological replications were used for each leaf sample with their respective two technical replicates. Means for control and IR stimulus treatments, at each evaluation time, followed by an asterisk (*), are significantly different (*p* ≤ 0.05) according to Tukey’s test. Means for NI and I plants, for each treatment, at each evaluation time, with a filled triangle (▲) are significantly different (*p* ≤ 0.05) according to an *F* test.

The expressions of *PAL3.1* and *LOX7* for non-inoculated plants were not affected by any treatments regardless of the evaluation time. The expressions of *CHIB1*, *PR1-A*, *PR10*, and *ACS* at 1 dai; *PAL2.1*, *CHIB1*, *ICS1*, *ICS2*, *JAR*, *ACO*, and *OPR3* at 3 dai; *PR1-A*, *ICS1*, *ICS2*, and *ETR1* at 5 dai; *PAL 2.1*, *ACS*, and *OPR3* at 10 dai; and *PR1-A*, *PR10*, *ICS1*, and *OPR3* at 15 dai were significantly higher, while expressions of *ETR1* and *OPR3* at 1 dai as well as *CHIB1* and *ACO* at 10 dai were significantly lower for IR stimulus treatment compared to the control treatment (Figure 12A,B).

#### 2.10.2. Comparing Control and IR Stimulus Treatments for Inoculated Plants

For inoculated plants, the expressions of *PAL2.1*, *CHIB1*, *PR1-A*, *PR10*, *ICS2*, *JAR*, *ACS*, *ACO*, and *OPR3* at 1 dai; *PAL3.1*, *CHIB1*, *LOX7*, *PR-1A*, *PR10*, *ICS1*, *ICS2*, *ETR*, *ACS*, and *OPR3* at 3 dai; *PAL2.1*, *PAL3.1*, *CHIB1*, *LOX7*, *PR1-A*, *PR10*, *ICS1*, *ICS2*, *JAR*, *ETR*, *ACS*, *ACO*, and *OPR3* at 5 and 10 dai as well as *PAL2.1*, *PAL3.1*, *CHIB1*, *LOX7*, *PR1-A*, *PR10*, *ICS1*, *ICS2*, *JAR*, *ETR*, *ACS*, and *OPR3* at 15 dai were significantly higher while expressions of *JAR* at 3 dai and *TEF-1α* at all evaluation times were significantly lower for IR stimulus treatment in comparison to the control treatment (Figure 12C,D).

#### 2.10.3. Comparing Non-Inoculated and Inoculated Plants for Control Treatment

For control treatment, the expressions of *PAL2.1*, *CHIB1*, and *PR10* at 1 dai; *PAL2.1*, *ICS2*, *JAR*, and *OPR3* at 3 dai; *PAL3.1*, *PR10*, *ICS1*, and *ICS2* at 5 dai as well as *LOX7*, *PR1-A*, and *PR10* at 15 dai were significantly higher for inoculated plants in comparison to non-inoculated plants. Conversely, the expressions of *PAL3.1*, *LOX7*, *PR-1A*, *ICS1*, and *ETR* at 1 dai; *LOX7* and *PR1-A* at 3 dai; *JAR*, *ETR*, *ACS*, and *ACO* at 5 dai; *PAL3.1*, *LOX7*, *PR-1A*, *ICS1*, *JAR*, *ETR*, *ACS*, and *ACO* at 10 dai as well as *ETR*, *ACS*, and *ACO* at 15 dai were significantly lower for inoculated plants compared to non-inoculated plants (Figure 12A–D).

#### 2.10.4. Comparing Non-Inoculated and Inoculated Plants for IR Stimulus Treatment

For IR stimulus treatment, the expressions of *PAL2.1*, *CHIB1*, *PR10*, *ICS2*, *JAR*, and *OPR3* at 1 dai; *PAL3.1*, *CHIB1*, *LOX7*, *PR10*, *ICS1*, *ICS2*, *ETR*, *ACS*, and *OPR3* at 3 dai; *PAL2.1*, *PAL3.1*, *CHIB1*, *LOX7*, *PR1-A*, *PR10*, *ICS1*, *ICS2*, *JAR*, *ETR*, and *OPR3* at 5 dai; *PAL2.1*, *CHIB1*, *LOX7*, *PR1-A*, *PR10*, *ICS1*, *ICS2*, *JAR*, *ETR*, *ACO*, and *OPR3* at 10 dai as well as *PAL2.1*, *PAL3.1*, *CHIB1*, *LOX7*, *PR1-A*, *PR10*, *ICS1*, *ICS2*, *JAR*, *ACS*, and *OPR3* at 15 dai were significantly higher for inoculated plants compared to non-inoculated plants. Conversely, the expressions of *PAL3*.1, *ICS1*, and *ETR* at 1 dai; *ACO* at 3 and 15 dai as well as *PAL3.1* and *ACS* at 10 dai were significantly lower for inoculated plants in comparison to non-inoculated plants (Figure 12A–D).

### 2.11. PCA

**Figure 13:**
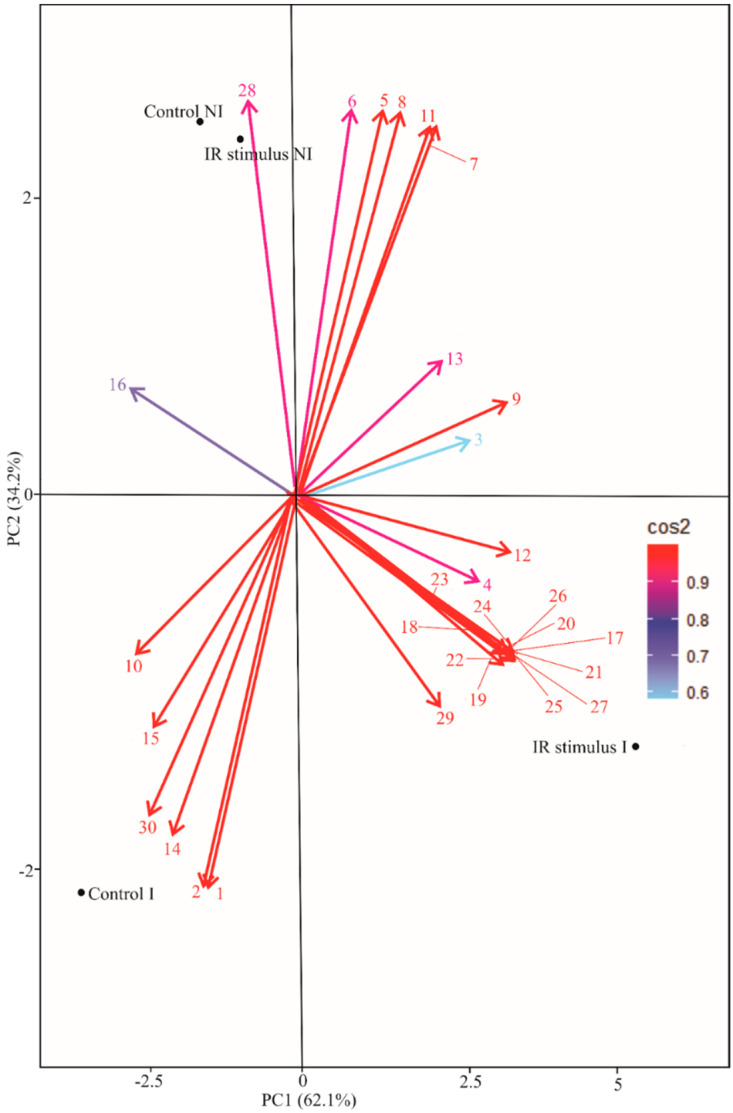
Score plots and loading values in the principal component analysis (PCA) for variables and parameters evaluated in soybean plants sprayed with water (control) or with induced resistance (IR) stimulus and non-inoculated (NI) or inoculated (I) with *Phakopsora pachyrhizi*. The numbers in the PCA are as follows: severity (1), area under disease progress curve (2), foliar concentrations of Ni and K (3 and 4, respectively), concentrations of photosynthetic pigments (5 and 6, respectively, to Chl *a* + *b* and carotenoids), parameters of chlorophyll *a* fluorescence [7, 8, 9, 10, and 11, respectively, to *F*_v_/*F*_m_, Y(II), Y(NPQ), Y(NO), and ETR], metabolites (12, 13, 14, 15 and 16, respectively, to TSP, LTGA derivatives, MDA, H_2_O_2_, and O_2_^•−^), and gene expression (17, 18, 19, 20, 21, 22, 23, 24, 25, 26, 27, 28, 29, and 30, respectively, to *PAL2.1*, *PAL3.1*, *CHIB1*, *LOX7*, *PR-1A*, *PR10*, *ICS1*, *ICS2*, *JAR*, *ETR1*, *ACS*, *ACO*, *OPR3*, and *TEF-1α*). Groups were generated from cluster analysis with complete linkage and Pearson distance. Data from variables and parameters used in the PCA were obtained 15 days after the inoculation of plants with *P*. *pachyrhizi* and also from NI plants at this same evaluation time.

Four clusters were generated (separate NI and I plants for control and IR stimulus treatments) based on the cluster analysis with complete linkage and Pearson distance. One principal component (PC) explained most data variation (PC1 = 61.3% and PC2 = 35.1%). The PC1 indicated negative scores for severity, AUDPC, Y(NO), MDA, H_2_O_2_, O_2_^•−^, *ACO*, and *TEF-1*α while positive scores were obtained for Ni, K, Chl *a* + *b*, Car, *F*_v_/*F*_m_, Y(II), Y(NPQ), ETR, TSP, LTGA derivatives as well as for the expression of some genes (*PAL2.1*, *PAL3.1*, *CHIB1*, *LOX7*, *PR-1A*, *PR10*, *ICS1*, *ICS2*, *JAR*, *ETR1*, *ACS*, and *OPR3*). For PC2, negative scores were obtained for severity, AUDPC, K, Y(NO), TSP, MDA, H_2_O_2_, and for the expression of some genes (*PAL2.1*, *PAL3.1*, *CHIB1*, *LOX7*, *PR-1A*, *PR10*, *ICS1*, *ICS2*, *JAR*, *ETR1*, *ACS*, *OPR3*, and *TEF-1*α), while positive scores were obtained for Ni, Chl *a* + *b*, Car, *F*_v_/*F*_m_, Y(II), Y(NPQ), ETR, LTGA derivatives, O_2_^•−^, and *ACO* (Figure 13).

## 3. Discussion

Soybean cultivars with race-specific resistance, or even those displaying the highest level of non-race-specific resistance that will impact some components of resistance (e.g., incubation period, latent period, and infectious period), are not available to growers [9,33]. On top of that, the capacity of *P. pachyrhizi* to become more sensitive to the currently used fungicides is an actual issue among soybean growers [33]. In these scenarios, the use of resistance inducers may become a great tool that could be used in an integrated rust management program. The present study brings biochemical, molecular, and physiological evidence for using an IR stimulus to boost soybean resistance against rust. Phosphites have great potential to induce plant defense reactions against pathogens causing a plethora of diseases, in addition to having a direct effect against mycelial growth and spore germination [30,34,35,36,37]. Interestingly, the IR stimulus used in this study was efficient in reducing the rust symptoms considering the lower severity, the leaflet tissues colonized by *P. pachyrhizi* (less *TEF-1α* expression), and the impaired reproduction, evidenced by the reduced number of uredinia containing a lower amount of urediniospores in the necrotic lesions formed on the leaflets. In addition, the IR stimulus helped reduce the cellular damage provoked by fungal infection in the leaflets by decreasing the pool of MDA, H_2_O_2_, and O_2_^•−^. The in vitro assay demonstrated the direct effect of the IR stimulus to reduce the germination of urediniospores from *P. pachyrhizi*. It is known that some IR stimuli (e.g., acibenzolar-S-methyl, azelaic acid, copper-polyphenolic compound, hexanoic acid, nickel, and phosphites formulated with different nutrients) have been able to inhibit mycelia growth or spore germination in vitro [3]. The mycelia growth of *Sclerotinia sclerotiorum* was significantly reduced by different types of phosphite [28,38,39]. Nickel was also efficient in reducing the germination of conidia from *Bipolaris oryzae* and urediniospores from *P. pachyrhizi*, as well as mycelia growth from *Exserohilum turcicum* [40,41]. The urediniospores from *P. pachyrhizi* showed shorter germ tubes after being exposed to a copper–polyphenolic compound as well as to azelaic and hexanoic acids [23,25,26]. The growth of germ tubes from urediniospores of *P. pachyrhizi* and *Puccinia emaculata* was greatly reduced by the phosphite of potassium in vitro [31]. Guo et al. [42] also reported that different rates of phosphite of potassium inhibited the mycelial growth of *Phytophthora sojae*.

Plants can absorb and translocate a large amount of nutrients sprayed to their shoots and use them to improve the basic physiological process that will result in better growth and yield, in addition to being used to mount defense reactions against infection by pathogens [37,43,44,45]. Increases in foliar concentrations of Ni and K for different crops in response to the foliar spray of products containing these nutrients have been reported in the literature [23,40,41,46]. The present study noticed higher foliar concentrations of Ni and K for infected and IR-stimulus-sprayed plants. Higher foliar K concentration for infected and IR-stimulus-sprayed plants helped to lower rust symptoms, considering the role played by this macronutrient in increasing the production of phenolics, improving photosynthesis (stomatal aperture and osmoregulation), as a co-factor of different enzymes, and in the synthesis of proteins [47]. On the other hand, Ni plays an important role in plant physiology, as it is associated with some enzymes that are important in scavenging some reactive oxygen species (superoxide dismutase and catalase) in stressed plant tissues, in addition to having a direct effect against fungal growth and spore germination [23,40,41,48]. In the soybean–*Phakopsora pachyrhizi* pathosystem, the foliar spraying of Ni reduced rust symptoms due to the higher expression of defense-related genes (e.g., chalcone isomerase, phenylalanine ammonia-lyase, and urease), the great production of lignin, higher *β*-1,3-glucanase activity, less oxidative damage (lower MDA, H_2_O_2_, and O_2_^•−^ concentrations) and less impairment of the photosynthetic apparatus (maintenance of Chl *a* fluorescence parameters and a great pool of photosynthetic pigments) [23].

Infection by pathogens of different lifestyles can seriously impair the functioning of the photosynthetic apparatus by reducing the pool of Chl *a* + *b* and carotenoids in plant tissues and negatively affecting the outcome of Chl *a* fluorescence parameters [49,50,51]. There are several studies reporting the potential of different pathogens of plants (e.g., *B. oryzae*, *E. turcicum*, *P. pachyrhizi*, *Pyricularia oryzae*, *S. sclerotiorum*, and *Septoria lycopersici*) to impair the photosynthetic performance of their hosts drastically and, consequently, contribute to disease symptom development [25,26,29,38,40,41,49,50,52,53]. In the present study, the infection of soybean leaflets by *P. pachyrhizi* severely impaired photosynthesis, as pictured by an expressive alteration of Chl *a* fluorescence [lower values for *F*_v_/*F*_m_, Y(II), Y(NPQ), and ETR along with higher Y(NO) values] parameters and the reduced pool of photosynthetic pigments (Chl *a* + *b* and carotenoids), suggesting the occurrence of photoinhibition, a reduction in the conversion of photochemical energy, and severe damage to the PSII. Rios et al. [50] highlighted the capacity of rust to significantly impair the photosynthetic apparatus of soybean plants, considering the lower values obtained for *F*_v_/*F*_m_ and Y(II) and the smooth increase in Y(NO). It is known that the application of some IR stimuli (e.g., acibenzolar-S-methyl, azelaic acid, hexanoic acid, products containing different nutrients mixed with polyphenolic compounds, picolinic acid, and phosphites) can attenuate the damage in the photosynthetic process and the pool of pigments caused by pathogenic infection [23,25,26,29,38,40,41,52]. In general, the symptoms of the diseases caused by these pathogens belonging to different lifestyles (e.g., biotrophic, hemibiotrophic, and necrotrophic) lower the influx of CO_2_ into the substomatal cavity on leaves due to either stomatal closure or destruction, biochemical restrictions on the mesophyll, and the reduced activity of ribulose bisphosphate carboxylase, which severely impact the photosynthetic apparatus. The leaves of tomato plants infected by *S. sclerotioum* and sprayed with a phosphite of manganese exhibited less impairment of the photosynthetic apparatus (increases in *F*_v_/*F*_m_, Y(II), and ETR values) and the preservation of Chl *a* + *b* and carotenoids [38]. In the present study, the spraying of IR stimulus greatly alleviated the damage caused by *P. pachyrhizi* at the physiological level. The values of *F*_v_/*F*_m_, Y(II), Y(NPQ), and ETR significantly increased. In contrast, the Y(NO) values decreased in infected and IR-stimulus-sprayed plants, confirming less photodamage, an efficient photochemical energy conversion, and the higher preservation of the PSII. Consistent with these findings, Picanço et al. [29] reported greater values for *F*_v_/*F*_m_, Y(II), and ETR associated with reduced Y(NO) values for soybean plants sprayed with phosphite combined with free amino acids and infected by *P. pachyrhizi*, which guaranteed less photodamage that ensured that adequate functionality of the photosynthetic apparatus was retained. The infection of soybean leaflets by *P. pachyrhizi* was negatively impacted with the spraying of a copper–polyphenolic compound, along with the preservation of photosynthesis [higher Y(II) and ETR values linked with reduced Y(NPQ) and Y(NO) values] and a greater pool of photosynthetic pigments [25]. The concentrations of Chl *a* + *b* (at the earlier and late stages of rust progress) and carotenoids (at an advanced stage of rust progress) were maintained at higher values for infected and IR-stimulus-sprayed plants, highlighting its efficiency in keeping plants with more robust photosynthetic machinery.

The ROS, mainly H_2_O_2_ and O_2_^•−^, is found in plant tissues of different crops infected by pathogens [54]. It is known that ROS can greatly impair cell physiology due to lipid peroxidation in the membranes as well as the degradation of photosynthetic pigments, proteins, and nucleic acid [37,49,55,56]. In some dicots, some types of phosphites promoted signaling pathways mediated by hormones or increased the production of H_2_O_2_ [31,57]. In the present study, the concentration and the histochemical accumulation of H_2_O_2_ and O_2_^•−^, along with MDA, a lipid peroxidation marker, were great for infected leaflets of water-sprayed plants due to intensive rust development. In contrast, infected and IR-stimulus-sprayed plants displayed less oxidative stress based on lower H_2_O_2_, O_2_^•−^, and MDA concentrations. According to Rodrigues et al. [25], the histochemical localization of H_2_O_2_ and O_2_^•−^ in the leaflets of soybean plants infected by *P. pachyrhizi* sprayed with a copper–polyphenolic compound was weakly detected. The genes involved in oxidoreductase activity (*AOX*) and the ROS pathway were strongly induced in switchgrass infected by *P. emaculata* [31].

To cope with infection by pathogens of different lifestyles, plants have developed a very efficient defense system that is formed by multilayered mechanisms and strategies, such as the modification of the cell-wall composition, the production of ROS, the expression of various defense-related genes (*PR-1A*, *PR10*, *PAL2.1*, and *PAL3.1*), and a high production of phenolics, flavonoids, and phytoalexins [54]. Phenolics and lignin precursors, originating from the phenylpropanoid pathway, are of pivotal importance for plant defense against pathogens due to their strong antimicrobial and antioxidant characteristics [58,59,60,61]. A large body of studies highlights the importance of phenolics and lignin in reducing disease symptoms in plants sprayed with different IR stimuli [25,26,29,40,62]. In the present study, TSP- and LTGA-derivative concentrations were greatly enhanced on infected leaflets of IR-stimulus-sprayed plants, which greatly helped to boost their resistance against infection by *P. pachyrhizi*. The spraying of phosphite combined with free amino acids helped to increase the pool of TSP and LTGA derivatives in the leaflets of soybean plants infected by *P. pachyrhizi* [29].

During SAR, a plethora of genes (e.g., *CHIB1*, *ICS1*, *ICS2*, *PAL1.1*, *PAL2.1*, *PR1*, and *PR2*) are promptly and strongly up-regulated with the involvement of SA and other molecules [54,63,64]. The SA originates from either phenylpropanoid or isochorismate pathways in which *PAL* and *ICS* play, respectively, an essential role in increasing the amount of this hormone [20,59,60]. In soybean, an array of molecules and IR stimuli (e.g., acibenzolar-S-methyl, azelaic acid, copper–polyphenolic compound, hexanoic acid, jasmonic acid, nickel, phosphite combined with free amino acids, and silicon) were reported to efficiently stimulate the expression of many defense-related genes (*PAL2.1*, *PAL3.1*, *ICS1*, *ICS2*, *PR-1A*, *PR10*, and *CHIB1*) involved in SAR [3,22,23,25,26,29]. In the present study, *PAL2.1* (at 3 and 10 dai), *ICS1* (at 3, 5, and 10 dai), and *ICS2* (at 3 and 5 dai) were up-regulated for non-infected and IR-stimulus-sprayed plants indicating the elicitation of host defense reactions. On the other hand, *PAL2.1*, *PAL3.1*, *ICS1*, and *ICS2* were strongly up-regulated during the time course of infection by *P. pachyrhizi* in IR-stimulus-sprayed plants, indicating the role played by these genes in the increased resistance of soybean plants. The expression of the genes mentioned above was strongly linked to the great pool of TSP and LTGA derivatives, as evidenced in infected leaflets of IR-stimulus-sprayed plants that displayed lower rust symptoms and fungus reproduction. The spraying of soybean plants with phosphites formulated with copper and manganese increased PAL activity, which had their resistance against rust boosted [32]. The up-regulation of *PR* genes in response to infection by pathogens plays an essential role in plant defense during SAR [54,65,66]. The *PR1* is a molecular marker for SAR [65,66]. In the present study, *PR-1A* and *PR10* were up-regulated for both non-infected and infected leaflets of IR-stimulus-sprayed plants, confirming a rise in their base resistance level. Interestingly, these genes were strongly up-regulated during the time course evaluated for the infected and IR-stimulus-sprayed plants and may be linked to reduced rust severity. Soybean plants sprayed with phosphite of K and infected by *P. pachyrhizi* displayed great *PR1* expression and less rust severity [31]. In addition, Guo et al. [43] reported a more robust up-regulation of *PR1* in leaves, stems, and roots of soybean plants infected by *P. sojae* after spraying them with phosphite of K. Chalcone isomerase, encoded by *CHIB1*, participates in the flavonoid pathway, leading to the biosynthesis of different antimicrobial compounds against pathogens [67]. In the present study, *CHIB1* was up-regulated for non-infected and IR-stimulus-sprayed plants only at 1 and 3 dai, while for infected and IR-stimulus-sprayed plants, a stronger up-regulation of this gene occurred during the entire time-course evaluated, confirming, therefore, the potential of this IR stimulus to boost soybean defense against infection by *P. pachyrhizi*. Rodrigues et al. [26] also reported the up-regulation of *CHIB1* in leaflets of soybean plants sprayed with a copper–polyphenolic compound and non-infected or infected by *P. pachyrhizi*.

Plants can express SAR or ISR in response to infection by pathogens from different lifestyles, without discarding that crosstalk between them may also occur at some point on their signal transduction pathways [3,15,54,68]. Different variables, e.g., the level of cultivar resistance, the type of the IR stimulus (rate and time of application), and the lifestyle of the pathogen and its level of aggressiveness, may modulate the response of plants against infection by pathogens, in terms of which pathway to be activated if mediated by SA or JA/ET [13,16,20,64]. In soybean–*P. pachyrhizi* interaction, several studies reported the activation of the JA/ET pathway by different types of IR stimuli (e.g., azelaic acid, calcium silicate, copper–polyphenolic compound, hexanoic acid, and jasmonic acid) [22,25,26]. In the present study, genes involved in the jasmonic acid (*JAR*, *LOX7*, and *OPR3*) and ethylene (*ETR1*, *ACS*, and *ACO*) pathways were strongly up-regulated in infected and IR-stimulus-sprayed plants at earlier and advanced stages of fungal infection. This finding confirms the participation of the JA/ET pathway in the soybean defense against rust after being exposed to the IR stimulus. On top of that, the IR-stimulus-sprayed plants not infected by *P. pachyrhizi* displayed an earlier up-regulation of *JAR*, *OPR3*, *ETR1*, *ACS*, and *ACO*, suggesting that these plants were primed. Gill et al. [32] reported a remarkable up-regulation of *OPR* in switchgrass infected by *P. emaculata* in response to phosphite of K. Tomato plants sprayed with phosphite combined with free amino acids and infected by *Septoria lycopersici* activated the JA/ET pathway (stronger expression of *ACO2*, *ACO3*, *ACO4*, *ACO5*, *LOX1.1*, *LOXB*, *LOXC*, and *PDF1.2*) and exhibited increased resistance against fungal infection [53]. It is tempting to assume that the IR stimulus used in the present study was efficient in priming plants for both SA and JA/ET signaling pathways and, therefore, contributed to reducing rust severity. According to Rodrigues et al. [27], both SA and JA/ET pathways were involved in the resistance of soybean plants against infection by *P*. *pachyrhizi* (great up-regulation of *URE*, *ICS2*, *CHIB1*, *PAL1.1*, *PAL2.1*, *PAL3.1*, *PR1-A*, *SABATH2*, *JAR1*, *PR10*, *MMP2*, *NAC19*, *ETR1*, and *OPR3*) after being elicited by hexanoic acid.

In conclusion, the biochemical, molecular, and physiological evidence in this study shows the potential of using the phosphite of K and Ni to boost soybean resistance against rust. It was evidenced in the PCA that infected and IR-stimulus-sprayed plants displayed remarkable differences from the infected and water-sprayed plants, considering the outcome of all variables and parameters. In short, infected and IR-stimulus-sprayed plants were more responsive against infection by *P. pachyrhizi* based on the different sets of defense responses [an earlier and stronger activation of genes involved in SA and JA/ET signaling pathways (indicating an interplay between these pathways and no advantage of one over the other)], lower cellular damage, a higher production of phenolics and lignin, the preservation of the photosynthetic apparatus, and great foliar concentrations of K and Ni). Together, these results open the way for using this IR stimulus as an eco-friendly alternative in integrated rust management to decrease yield losses caused by rust and improve the quality of soybean grains.

## 4. Material and Methods

### 4.1. In Vitro Assay

Different volumes of a stock solution (80 mL/L) of Blindage Ni^®^ [nickel (0.5% Ni and 7 g/L), potassium (20% K_2_O and 280 g/L), and phosphorous acid (500 g/L); Unity Agro, Curitiba, Brazil] were mixed with 1 mL of a suspension of *P*. *pachyrhizi* urediniospores (10^5^ urediniospores/mL) to obtain the final concentrations of 2, 5, 7, 10, and 15 mL/L. A total of 100 µL of urediniospores’ suspension (10^5^ urediniospores/mL) containing the different concentrations of Blindage^®^ was transferred to a glass slide and covered with a coverslip. A suspension of urediniospores without Blindage^®^ solution corresponded to the control treatment. The glass slides were transferred to a growth chamber (25 °C and 12 h light/12 h dark photoperiod). Each glass slide received 5 μL of lactophenol after 12 h to stop urediniospores’ germination. One hundred urediniospores were randomly examined in each glass slide under a light microscope (Carl Zeiss AxioImager A1; ZEISS AG, Oberkochen, Germany) at 40× magnification using the bright field. The images of the details for the urediniospores’ germination were acquired digitally (model AxioCam HR and Axion Vision software v. 4.8.1; Zeiss AG, Oberkochen, Germany). A urediniospore with a germ tube larger than its diameter was considered germinated. The percentage of the urediniospores’ germination was calculated for the replications of each treatment.

### 4.2. Growth of Soybean Plants

A total of six soybean seeds from cultivar DS5916IPRO [susceptible to *P. pachyrhizi*; https://www.brevant.com.br) were sown in each plastic pot containing 2 Kg of a 1:1 mixture of soil and substrate (Tropstrato^®^, Vida Verde, Mogi Mirim, Brazil). After germination, a total of four seedlings were left per pot. The plants in each pot were fertilized weekly with 100 mL of nutrient solution by Clark [69], with some modifications, which consisted of 1.04 mM Ca(NO_3_)_2_⋅4H_2_O, 1 mM NH_4_NO_3_, 0.8 mM KNO_3_, 0.6 mM MgSO_4_⋅7H_2_O, 0.069 mM KH_2_PO_4_, 0.931 mM KCl, 19 µM H_3_BO_3_, 2 µM ZnSO_4_⋅7H_2_O, 7 µM MnCl_2_⋅4H_2_O, 0.6 µM Na_2_MoO_4_⋅4H_2_O, 0.5 µM CuSO_4_⋅5H_2_O, 90 µM FeSO_4_⋅7H_2_O, and 90 mM ethylenediaminetetraacetic acid disodium (EDTA). Plants were grown in a greenhouse with a temperature of 25 ± 2 °C, a relative humidity of 70 ± 5%, and a natural photosynthetically active radiation (PAR) of 915 ± 12 μmol photons m^−2^ s^−1^ measured at midday.

### 4.3. Application of Blindage^®^

Soybean plants (V4 growth stage; ≈30 days after seedlings’ emergence) were sprayed with a solution of Blindage^®^ (7.5 mL/L, 5 mL of solution per plant) using a VL Airbrush atomizer (Paasche Airbrush Co., Chicago, IL, USA). According to the criteria proposed by Kesel et al. [16], this treatment will be referred to as IR stimulus. The IR stimulus solution was prepared using deionized water. Plants sprayed with water served as the control treatment.

### 4.4. Inoculation of Soybean Plants with P. pachyrhizi

Plants were inoculated with a suspension of 10^5^ urediniospores of *P. pachyrhizi*/mL prepared with gelatin (0.5% *w*/*v*) and Tween 80 (25 μL/L) by using a VL Airbrush atomizer at 48 h after being sprayed with water or IR stimulus. After inoculation, plants were kept in a mist chamber at 25 °C for 16 h under darkness. After this period, plants were transferred to a greenhouse (a temperature of 25 ± 2 °C, a relative humidity of 75 ± 5%, and a natural PAR of 932 ± 20 μmol photons m^−2^ s^−1^ measured at midday) until the end of the experiments. Plants non-inoculated with *P. pachyrhizi* were kept in a different mist chamber and greenhouse under the same environmental conditions mentioned above.

### 4.5. Experimental Design

For the in vitro assay, the experiment was arranged in a completely randomized design with six treatments (control and five concentrations of IR stimulus) and ten replications. Each replication corresponded to one glass slide. A 2 × 2 factorial experiment, consisting of plants sprayed with water (control) and IR stimulus and non-inoculated or inoculated with *P. pachyrhizi*, was arranged in a completely randomized design with six replications per evaluation time to assess rust severity, as well as to determine the foliar concentrations of Ni and K. Another 2 × 2 factorial experiment with the same factors mentioned above and ten replications per evaluation time was carried out to evaluate the fluorescence of chlorophyll *a* fluorescence parameters and to quantify the foliar concentration of pigments. Leaf samples for the scanning electron microscopy (SEM) and histochemical observations, as well as for the biochemical and gene expression analysis, were obtained from another 2 × 2 factorial experiment with the same factors described above and ten replications per evaluation time. All experiments were repeated once.

### 4.6. Evaluation of ASR Severity

The leaflets of the second and third leaves, from base to top, of each plant per replication (six replications in a total of 24 plants and 48 leaves) of each treatment were used to evaluate SR severity at 7, 9, 11, 13, and 15 days after inoculation (dai) according to the diagrammatic scale proposed by Franceschi et al. [70]. The area under disease progress curve (AUDPC) for each leaflet per the leaf of each plant from the replications of each treatment was calculated using the trapezoidal integration of disease progress curves [71]. At 15 dai, the second and third leaves of each plant per replication of each treatment were collected and scanned at 600 dpi resolution. The images were processed using the software QUANT Version 1.0 to obtain the values of final SR severity according to Fagundes-Nacarath et al. [40].

### 4.7. Determination of Foliar Nickel (Ni) and Potassium (K) Concentrations

The leaflets of the second and third leaves, from base to top, of each plant per replication (six replications in a total of 24 plants and 48 leaves) of each treatment obtained at 15 dai were washed in deionized water and dried in a drying oven with forced ventilation for 48 h. The foliar Ni and K concentrations were determined by nitric–perchloric digestion and inductively coupled plasma-optical emission spectrometry (ICP-OES).

### 4.8. Processing Leaf Samples for SEM

A total of 50 fragments (≈5 mm^2^) were randomly obtained from the leaflets of the second and third leaves, from base to top, of each plant per replication (six replications in a total of 24 plants and 48 leaves) of each treatment at 15 dai. The fragments were carefully transferred to glass vials containing 10 mL of fixative [3% (*v*/*v*) glutaraldehyde and 2% paraformaldehyde (*v*/*v*) in 0.1 M sodium cacodylate buffer (pH 7.2)], stored at 4 °C for 10 days. Leaflet fragments were washed with sodium cacodylate buffer (0.1 M), dehydrated in ethanol, and subjected to critical point drying using CO_2_ (model CPD 030; Electron Microscopy Sciences, Hatfield, PA, USA). Five leaf fragments were mounted on aluminum stubs, sputter-coated with gold (model FDU 010; Electron Microscopy Sciences, Hatfield, PA, USA), and uredinia containing urediniospores of *P*. *pachyrhizi* were observed in an ZEISS LEO SEM (model 1430VP; ZEISS AG, Oberkochen, Germany) operating at 10 kV and a working distance of 15 mm.

### 4.9. Imaging and Quantification of Chlorophyll (Chl) a Fluorescence Parameters

The images and parameters of Chl *a* fluorescence in the leaflets of the second leaf, from base to top, of each plant per replication (ten replications in a total of 40 plants and 40 leaves) of each treatment were obtained using the MAXI version of the Imaging-PAM fluorometer and the Imaging Win software version 1.0 (Heinz Walz GmbH, Effeltrich, Germany) at 7, 11, and 15 dai. The leaflets of the second leaf from non-inoculated plants were also evaluated at these evaluation times. The procedures described by Picanço et al. [72] were used to obtain the images of Chl *a* fluorescence parameters and their further quantification.

### 4.10. Determining Photosynthetic Pigments Concentration

Five leaf disks (1 cm^2^ each) were obtained from the leaflets of the second leaf, from base to top, of each plant per replication (ten replications in a total of 40 plants and 40 leaves) of each treatment at 7, 11, and 15 dai. The disks were immersed in glass tubes containing 5 mL of saturated dimethyl sulfoxide solution and calcium carbonate (5 g/L), kept in the dark at room temperature for 24 h, and the absorbances of the extracts were read at 480, 649, and 665 nm to determine the concentrations of Chl *a*, Chl *b*, and carotenoids according to Picanço et al. [72].

### 4.11. Histochemical Detection of Lipid Peroxidation, Membrane Damage, Hydrogen Peroxide (H_2_O_2_), and Superoxide Anion Radical (O_2_^•−^)

The leaflets of the second leaf, from base to top, of each plant per replication (ten replications in a total of 40 plants and 40 leaves) of each treatment were collected from both non-inoculated and inoculated plants at 15 dai. Lipid peroxidation and membrane damage in the leaflets were visualized using the reagents of Schiff [73] and Evans blue [74]. The leaflets were randomly transferred to glass vials containing 50 mL of either Schiff (10% *v/v* prepared in deionized water) or Evans blue (0.025% *w*/*v* prepared in 100 μM of CaCl_2_, pH 5.6) reagents (five leaflets per glass vial for each reagent) at 2 and 12 h, respectively. For H_2_O_2_ detection, leaflets were randomly placed in glass vials (five leaflets per glass vial) containing 25 mL of 3,3′-diaminobenzidine tetrahydrochloride solution (1 mg/mL) (Sigma-Aldrich, São Paulo, Brazil) and kept in the dark at 25 °C for 12 h. For O_2_^•−^ detection, leaflets were randomly placed in glass vials (five leaflets per glass vial) containing 50 mL of nitro blue tetrazolium (0.1%) solution (Sigma-Aldrich, São Paulo, Brazil) prepared in potassium phosphate buffer (10 mM, pH 6.8) over 24 h. The leaflets were cleared in boiling aqueous ethanol (80%) for 80 min until pink, blue, brown, and blue spots were noticed, confirming the presence of lipid peroxidation, membrane damage, H_2_O_2_, and O_2_^•−^, respectively.

### 4.12. Biochemical Assays and Gene Expression Using Quantitative Real-Time PCR

The second and third leaves, from base to top, of each plant per replication (ten replications in a total of 80 plants and 80 leaves) of each treatment were collected at 1, 3, 5, 10, and 15 dai from both non-inoculated and inoculated plants. Leaf samples were kept in liquid nitrogen during sampling and stored at −80 ºC until further analysis.

#### 4.12.1. Malondialdehyde (MDA) Concentration

Oxidative damage in the leaflet tissues was estimated based on the concentration of total 2-thiobarbituric acid (TBA) reactive substances and expressed as equivalents of MDA [75]. Leaflet tissue (0.1 g) was ground into a fine powder using a vibration ball mill (Retsch, Haan, Germany) with liquid nitrogen and homogenized in 2 mL of 0.1% (*w*/*v*) trichloroacetic acid (TCA) solution in an ice bath. The homogenate was centrifuged at 12,000× *g* for 15 min at 4 °C. After centrifugation, a total of 250 µL of the supernatant was reacted with 750 µL of TBA solution (0.5% in 20% TCA) for 60 min in a boiling water bath at 95°C. After this period, the reaction was stopped in an ice bath. The samples were centrifuged at 10,000× *g* for 10 min and the specific absorbance was determined at 532 nm. The non-specific absorbance was estimated at 600 nm and subtracted from the specific absorbance value and the extinction coefficient of 155 mM^−1^ cm^−1^ was used to calculate the MDA concentration.

#### 4.12.2. Concentrations of H_2_O_2_ and O_2_^•−^

Leaflet tissue (0.1 g) was ground into a fine powder described above and homogenized in 2 mL of 0.1% (*w*/*v*) of TCA. The homogenate was centrifuged at 12,000× *g* for 15 min at 4 °C. The supernatant was added to a reaction mixture containing 10 mM potassium buffer (pH 7.0) and 1 M of iodide solution and incubated for 10 min. The H_2_O_2_ concentration was determined based on the oxidized product formed at 390 nm [76]. A standard curve of H_2_O_2_ (Sigma-Aldrich, São Paulo, Brazil) was used to determine H_2_O_2_ concentration. Leaflet tissue (0.2 g) was ground as described above and homogenized in 2 mL of a solution containing 100 mM sodium phosphate buffer (pH 7.2) and 1 mM sodium diethyldithiocarbamate. The homogenate was centrifuged at 22,000× *g* for 20 min at 4 °C and the supernatant was used to determine O_2_^•−^ concentration according to Chaitanya and Naithani [77].

#### 4.12.3. Concentrations of Total Soluble Phenols (TSP) and Lignin-Thioglycolic Acid (LTGA) Derivatives

Leaflet tissue (0.1 g) was ground into a fine powder as described above and homogenized in 1 mL of 80% (*v*/*v*) methanol solution. The crude extract was shaken at 300 rpm at 25 °C for 2 h and the mixture was centrifuged at 17,000× *g* for 30 min. The TSP concentration was determined in the methanolic extract and the pellet was used to determine the LTGA derivative concentration according to Tatagiba et al. [78].

#### 4.12.4. Genes Expression

Leaflet tissue (0.1 g) was ground into a fine powder as described above. The fine powder was used for RNA extraction using Trizol (Invitrogen^®^, São Paulo, Brazil). Contamination by DNA was removed with RQ1 RNase-Free DNase (Promega, São Paulo, Brazil). The quality and integrity of the RNA were verified by 1.2% agarose gel electrophoresis and the amount of RNA was measured in a Qubit fluorometer using Qubit RNA HS Assay Kit (Invitrogen). Single-stranded cDNAs were synthesized by reverse transcription using 5 μg of total RNA with oligo(dT) primers in a final volume of 20 μL using the SuperScript First-Strand Synthesis System for RT-PCR (Invitrogen^®^). The qRT-PCR was performed on a Bio-Rad (Hercules, CA, USA) CFX Real-Time Thermal Cycler using SYBR Green PCR Master Mix according to the manufacturer’s recommendations. All reactions were duplicated and the relative expression values for each gene to be investigated were calculated using the 2^−ΔΔCt^ method [79]. An expression analysis of genes encoding for phenylalanine ammonia-lyase (*PAL2.1* and *PAL3.1*), chalcone isomerase (*CHIB1*), lipoxygenase (*LOX7*), pathogenesis-related protein 1 (*PR1-A*), pathogenesis-related protein 10 (*PR10*), isochorismate synthase (*ICS1* and *ICS2*), jasmonic acid-amino synthetase (*JAR*), ethylene receptor 1 (*ETR1*), 1-aminocyclopropane-1-carboxylic acid synthase (*ACS*), 1-aminocyclopropane-1-carboxylic acid oxidase (*ACO*), and 12-oxophytodienoic acid reductase 3 (*OPR3*) was carried out using specific primer sequences (Table 2). The expression of *TEF-1α*, corresponding to the translation elongation factor 1α of *P. pachyrhizi*, was quantified to confirm the presence of the fungus in the leaflet tissues. The Ubiquitin-3 (*UBIQ*) and glyceraldehyde-3-phosphate dehydrogenase (*GAPDH*) genes were used as references for normalization according to Mortel et al. [80].

### 4.13. Data Analysis

Data from urediniospores’ germination were subjected to analysis of variance (ANOVA) and means were compared by Tukey’s test (*p* ≤ 0.05). For other variables and parameters, data were subjected to ANOVA and comparisons between control and IR stimulus treatments as well as between non-inoculated and inoculated plants were made using the *F* test (*p* ≤ 0.05). Data were checked for normality and homogeneity of variance before ANOVA. The procedures described by Moore and Dixon [81] were followed to combine the data from the variables and parameters evaluated from the repeated experiments. The Minitab Statistical software version 21.4.2 was used for the statistical analysis mentioned above [82]. Data from all variables and parameters obtained from control and IR stimulus treatments for non-inoculated and inoculated plants at 15 dai were used for principal component analysis (PCA) using the software R version 4.4.2 [83].

## Figures and Tables

**Table 1:** The analysis of variance for the effects of products (P), plant inoculation (PI), and the interaction P × PI for urediniospores’ germination (UG), soybean rust (SR) severity, area under disease progress curve (AUDPC), foliar nickel (Ni) and potassium (K) concentrations, the parameters of chlorophyll (Chl) *a* fluorescence [maximum PSII quantum efficiency (*F*_v_/*F*_m_), photochemical yield (Y(II)), yield for dissipation by down-regulation (Y(NPQ)), the yield for other non-photochemical (non-regulated) losses (Y(NO)) and electron transport rate (ETR), concentrations of total chlorophyll *a* + *b* (Chl *a + b*), carotenoids (Car), total soluble phenolics (TSP), lignin-thioglycolic acid (LTGA) derivatives, malondialdehyde (MDA), hydrogen peroxide (H_2_O_2_), and the superoxide anion radical (O_2_^•−^), as well as the expression of genes coding for phenylalanine ammonia-lyase (*PAL2.1* and *PAL3.1*), chalcone isomerase (*CHI1B1*), lipoxygenase (*LOX7*), pathogenesis-related protein 1 (*PR-1A*), pathogenesis-related protein 10 (*PR10*), isochorismate synthase (*ICS1* and *ICS2*), jasmonic acid-amino synthetase (*JAR1*), ethylene receptor 1 (*ETR1*), 1-aminocyclopropane-1-carboxylic acid oxidase (*ACO*), 1-aminocyclopropane-1-carboxylic synthase (*ACS*), 12-oxophytodienoic acid reductase 3 (*OPR3*), and the translation elongation factor 1α of *Phakopsora pachyrhizi* (*TEF-1α*).

Variables/Parameters	IR Stimulus	PI	IR Stimulus × PI
UG	**<0.001**	-	-
SR severity	**<0.001**	-	-
AUDPC	**<0.001**	-	-
Ni	**<0.001**	0.975	0.975
K	**<0.001**	**0.010**	**0.044**
*F*_v_/*F*_m_	0.385	0.986	**0.041**
Y(II)	**<0.001**	**0.030**	**<0.001**
Y(NPQ)	**<0.001**	**<0.001**	**<0.001**
Y(NO)	**<0.001**	**0.004**	**<0.001**
ETR	**<0.001**	**<0.001**	**<0.001**
Chl *a* + *b*	**0.018**	**<0.001**	**0.007**
Car	**<0.001**	**<0.001**	**<0.001**
MDA	**<0.001**	**<0.001**	**<0.001**
H_2_O_2_	**<0.001**	**<0.001**	**0.029**
O_2_^•−^	**0.026**	**0.006**	**0.021**
TSP	**0.006**	**0.013**	**<0.001**
LTGA derivatives	**<0.001**	0.572	**<0.001**
*PAL2.1*	**0.006**	**0.006**	**0.006**
*PAL3.1*	**<0.001**	0.102	0.099
*CHIB1*	**<0.001**	**<0.001**	**<0.001**
*LOX7*	**0.002**	**0.003**	**0.002**
*PR-1A*	**0.031**	**0.032**	**0.032**
*PR10*	**<0.001**	**<0.001**	**<0.001**
*ICS1*	**<0.001**	**0.013**	**0.014**
*ICS2*	**0.046**	**0.043**	**0.046**
*JAR1*	0.683	**0.002**	0.434
*ETR1*	**<0.001**	**<0.001**	**<0.001**
*ACS*	**<0.001**	0.075	**<0.001**
*ACO*	**<0.001**	0.289	**0.004**
*OPR3*	**<0.001**	**<0.001**	**<0.001**
*TEF-1α*	**<0.001**	-	-

Bold values are significant at *p* ≤ 0.05.

**Table 2:** Primer sequences were used to study the expression of the following genes: phenylalanine ammonia-lyase (*PAL2.1* and *PAL3.1*), chalcone isomerase (*CHI1B1*), lipoxygenase (*LOX7*), pathogenesis-related protein 1 (*PR-1A*), pathogenesis-related protein 10 (*PR10*), isochorismate synthase (*ICS1* and *ICS2*), jasmonic acid-amino synthetase (*JAR1*), ethylene receptor 1 (*ETR1*), 1-aminocyclopropane-1-carboxylic acid oxidase (*ACO*), 1-aminocyclopropane-1-carboxylic synthase (*ACS*), 12-oxophytodienoic acid reductase 3 (*OPR3*), glyceraldehyde 3-phosphate dehydrogenase (*GAPDH*), and Ubiquitin-3 (*UBIQ*) from soybean, as well as the translation elongation factor 1α from *Phakopsora pachyrhizi* (*TEF-1α*).

Genes	GenBank	Primer Sense 5′-3′	Primer Antisense 5′-3′
*PAL2.1*	*Glyma.10G058200*	ATCTCCCTCCACTCACCATA	GTTCAAGGGGTCATTAGCAC
*PAL3.1*	*Glyma.02G309300*	TGCTCTTCAGAAGGAAATGGT	GTTGCTGATTTAGGCAGTGT
*CHI1B1*	*Glyma.20G2416001*	GTTTCCCCTGCTTTGAAAGAGA	GGATTGGCCTCTAACTCTTTGAAG
*LOX7*	*Glyma.13G347800*	ACAAGCTAGGCACAACAAAA	TTGTTTCCTCCGATGATTCCAA
*PR-1A*	*Glyma.09G040500*	GCACTACACACAGGTCGTTTGG	CCTCCGTTATCACATGTCACTTTG
*PR10*	*Glyma.07G243651v4*	AAATCAACTCCCCTGTGGCTC	CCACCATTTCCCTCAACGTTT
*ICS1*	*Glyma.01G104100*	GAAACAGTACAGTCCCTGCT	TGTGGCTGGGAAAAGAAAAC
*ICS2*	*Glyma.03G070600*	GCAACATCCTCGTACCTCTT	CTCTCTGCAACCGTTCATTG
*JAR1*	*Glyma.07G057900*	AGCCGTATGGTTGTGTTGTTC	TGCAGCATTGGGATTGGAGT
*ETR1*	*Glyma.19g40090*	ATGGATGCCTTCAAGAAGTGG	GCACATATCTTCCCACAAGAGG
*ACS*	*Glyma.05g36250*	CTCTTAACCTTCATTCTTGCTAACC	TTGCTTCTGCTTCTTTGTATGC
*ACO*	*Glyma.14g05350*	CCAATGCGCCATTCCATTGTTG	TGAGGCTACGGACATTCTGGTC
*OPR3*	*Glyma.13G109800*	GTGTATCAGCCTGGTGGG	GTGTATCAGCCTGGTGGG
*GAPDH*	*Glyma.04G193500*	AAGGGTGGTGCAAAGAAGGT	TCTGGCTTGTACTCGTGCTC
*UBIQ*	*Glyma.20g141600*	TGTAATGTTGGATGTGTTCCC	GGGACACAATTGAGTTCAACA
*TEF-1α*	*Glyma.07G060900*	ATTCGAAGCCGGTATTTCTAAAG	CCACTTGGTTGTGTCCATCTTAT

## Data Availability

The datasets generated for this study are available upon request from the corresponding author.
